# Designing A Blockchain-Empowered Telehealth Artifact for Decentralized Identity Management and Trustworthy Communication: Interdisciplinary Approach

**DOI:** 10.2196/46556

**Published:** 2024-09-25

**Authors:** Xueping Liang, Nabid Alam, Tahmina Sultana, Eranga Bandara, Sachin Shetty

**Affiliations:** 1 Florida International University Miami, FL United States; 2 Troy University Troy, AL United States; 3 Old Dominion University Suffolk, VA United States

**Keywords:** telehealth, blockchain, security, software, proof of concept, implementation, privacy

## Abstract

**Background:**

Telehealth played a critical role during the COVID-19 pandemic and continues to function as an essential component of health care. Existing platforms cannot ensure privacy and prevent cyberattacks.

**Objective:**

The main objectives of this study are to understand existing cybersecurity issues in identity management and trustworthy communication processes in telehealth platforms and to design a software architecture integrated with blockchain to improve security and trustworthiness with acceptable performance.

**Methods:**

We improved personal information security in existing telehealth platforms by adopting an innovative interdisciplinary approach combining design science, social science, and computer science in the health care domain, with prototype implementation. We used the design science research methodology to implement our overall design. We innovated over existing telehealth platforms with blockchain integration that improves health care delivery services in terms of security, privacy, and efficiency. We adopted a user-centric design approach and started with user requirement collection, followed by system functionality development. Overall system implementation facilitates user requirements, thus promoting user behavior for the adoption of the telehealth platform with decentralized identity management and an access control mechanism.

**Results:**

Our investigation identified key challenges to identity management and trustworthy communication processes in telehealth platforms used in the current health care domain. By adopting distributed ledger technology, we proposed a decentralized telehealth platform to support identity management and a trustworthy communication process. Our design and prototype implementation using a smart contract–driven telehealth platform to provide decentralized identity management and trustworthy communication with token-based access control addressed several security challenges. This was substantiated by testing with 10,000 simulated transactions across 5 peers in the Rahasak blockchain network. The proposed design provides resistance to common attacks while maintaining a linear time overhead, demonstrating improved security and efficiency in telehealth services. We evaluated the performance in terms of transaction throughput, smart contract execution time, and block generation time. To create a block with 10,000 transactions, it takes 8 seconds on average, which is an acceptable overhead for blockchain-based applications.

**Conclusions:**

We identified technical limitations in current telehealth platforms. We presented several design innovations using blockchain to prototype a system. We also presented the implementation details of a unique distributed architecture for a trustworthy communication system. We illustrated how this design can overcome privacy, security, and scalability limitations. Moreover, we illustrated how improving these factors sets the stage for improving and standardizing the application and for the wide adoption of blockchain-enabled telehealth platforms.

## Introduction

Since the COVID-19 pandemic, the world has seen a dramatic shift toward social distancing efforts with the help of technological tools. Technology-enabled social distancing efforts have led to the wide adoption of work-from-home solutions, virtual meeting places including classrooms, financial service delivery, and health service delivery. A recent guidance regarding operation management from the US Centers for Disease Control and Prevention (CDC) indicates that telehealth services should be optimized when available and appropriate. Government health authorities from other developed and developing countries have issued similar directives. Moreover, health service organizations, such as hospital systems and insurance providers, have started to promote telehealth services to minimize physical traffic in medical facilities. With these recommendations and advancements, health care service use via telehealth services has seen a radical upshift, and it is expected that this trend will be sustained in the postpandemic era. The large-scale adoption of telehealth services within a short time comes with several social and technical concerns. Telehealth services overall are understudied, with complex, highly person-oriented, and often knowledge-intensive services, which pose difficulty in generating successful and sustainable business models [1]. Moreover, telehealth services pose privacy and identity protection threats to users that hinder telehealth’s successful adoption.

Personal privacy is a significant research stream [2,3]. Earlier studies have shown that user’s privacy concerns can be handled by reducing computer anxiety and increasing the incentive to disclose [4-6]. Later studies have shown more focus on protecting digital identity and trust to reduce privacy concerns, which can be managed well by increasing the trust of online services and increasing the protection of digital identity through anonymity. Sutanto et al [7] proposed an IT solution that delivers a personalized service but avoids the third-party use of personal information and thus increases the process and content gratification. As a decentralized solution to digital identity management and identity verification operations, blockchain technology, a consensus-based public or private ledger system, has emerged [8]. By considering the benefits of blockchain, such as user’s control over information and decentralized security features, various organizations (ie, the Government of Canada, the United Nations, etc) have started looking into the use case to enhance user privacy by decentralizing user information control [9-11].

Considering that blockchain integration into the health care industry could significantly transform traditional services with its security features and capabilities, previous research [12] has investigated blockchain adoption in the tracking of medical supplies and infected patients. The data can be handled instantly without any intermediary processing, thus improving efficiency, and data manipulation is impossible owing to the transparent nature of blockchain networks, which can further prevent misinformation [13] and preserve privacy. With the adoption of blockchain, data consistency and immutability can be assured along the data-sharing process, thus providing the capability of self-validation for each data record collected and uploaded. Most importantly, patients can limit personal information exposure under their own control. Fragmented records are easy to merge and integrate without any third-party involvement. Patients can own their health records, such as telehealth visits and immunization records, for future reference or verification.

State-of-the-art telehealth has emerged as a solution to overcome the challenges of an increasing demand for health care, an increasing demand for care outside of the hospital, a demand for efficiency and individualization, and a shortage of health care personnel to attend to an increasing number of patients [14]. Studies have shown that there is a positive relationship between telehealth and clinical care outcomes [15]. Although current research mostly focuses on the vital sign parameter monitoring solutions of telehealth [14], extant literature provides insights into which technology of telehealth is more efficacious. According to Dellifraine Jami and Dansky [15], video technology is more efficient among telehealth technologies because of its real-time interactivity. Moreover, studies have shown a relationship between different disease categories and telehealth technology. In a meta-analysis, Dellifraine Jami and Dansky [15] revealed that telehealth plays a positive role in improving the condition of psychiatric patients and plays a moderately positive role in heart failure outcomes. Additionally, no relationship was found between telehealth and diabetes outcomes. However, there are still challenges in combining incompatible information systems used by different stakeholders.

After 2010, telehealth solutions started to grow rapidly [16]. Telehealth is now addressing issues of chronic and episodic conditions along with acute conditions. Moreover, studies have shown that telehealth is an effective solution for mental health patients [17]. It has been found that telehealth solutions help in reducing health care costs, and the migration of telehealth has been seen from hospitals and satellite clinics to homes and mobile devices [16]. However, there are limitations of the current telehealth solutions that need to be addressed by future solutions. These limitations include limited insurance coverage of telehealth, lower quality of patient-physician relationships, different legal issues for different states, and a lack of trust from patients and unwillingness from clinicians [16]. Moreover, telehealth can be an effective solution during emergencies. Through telehealth, it is possible to respond quickly to any disaster. However, it is a matter of regret that still telehealth solutions are underutilized in emergency situations [18]. In the recent COVID-19 pandemic, we saw an emerging demand for telehealth in emergencies. There were restrictions on face-to-face interactions because of the highly infectious COVID-19. Therefore, telemedicine can provide medical advice and solutions in this regard. However, there is still training needed from the clinician’s side [19]. Moreover, a lack of trust remains a challenge. To overcome this challenge, the literature suggests that telehealth should be implemented proactively rather than reactively [19]. However, studies have also reported that there are risks of fake providers of telehealth in an emergency [18].

In information systems, discipline, collection, unauthorized secondary use, improper access, and errors are considered as the dimensions of information privacy [20]. Malhotra et al [21] have indicated that control, awareness, and collection of personal information can reflect the privacy concerns of an individual. It is identified that privacy concerns are dependent on computer anxiety [6]. This computer anxiety construct indicates that users are experiencing discomfort about the digitization of their personal life and society. Even though privacy protection is associated with positive practices, users are also motivated to share information about themselves if there are financial gains and conveniences offered by online service providers [4]. An experimental study found that (1) a clear privacy statement can reduce privacy concerns, but a security seal cannot; (2) monetary incentive influences information disclosure; and (3) information requests negatively influence information disclosure [5]. It is also found that perceived anonymity of self can reduce privacy concerns [22].

An IT solution [7] has been proposed that delivers a personalized service but avoids the third-party usage of personal information and thus increases the process and content gratification. Another study has shown that to balance the beneficial use of data and individual privacy in the era of big data, policymakers must address some of the most fundamental concepts of privacy, such as reducing the collection of personally identifiable information, increasing individual control, and implementing the principles of data minimization and purpose limitation policies [23]. An enhanced Antecedent-Privacy Concern-Outcome model [24] has shown that privacy experiences, awareness, personality, demographic differences, culture, and climate are the antecedents of privacy concern. Two recent studies have reflected on the data sharing and privacy aspects surrounding this [25,26]. According to Son and Kim [27], a major consequence of privacy concerns is the user’s privacy-protective behavior. Privacy-protective behavior can be defined as information provision (refusal and misinterpretation), private action (removal and negative word-of-mouth), and public action (direct complaint and indirect complaint).

On the other hand, the term “digital identity” has evolved through the process of digitization and the internet. Users, by their internet browsing history and footprint, educate others about who they are, what they do, and especially what they think. With the construction process, users’ digital footprints create their “digital identity.” In other literature, the term digital personhood is also used. Digital personhood refers mainly to the projected status in the online environment [28]. Windley [29] argues that the management and control of user’s digital identity, trust, and privacy are the fundamental concerns for both users and service providers in the current internet economy.

Prior telehealth platforms are exposed to certain levels of data privacy risks, lack of traceability, centralized control, and lack of data provenance. The research objective of this study is to develop a blockchain-assisted, smart contract–driven telehealth platform that is trustworthy and capable of preventing various cyberattacks. To achieve this objective, we followed the design science research (DSR) paradigm to guide the development of the proposed IT artifact [30] and adopted a user-centric design methodology. Based on that, we further analyzed system requirements and backend requirements to retrieve the design methodology. This combination of analysis helps us retrieve the design principles (DPs) and design features provided by blockchain integration. Experimental results reveal that the proposed design and implementation are baked with security considerations at an acceptable level of performance in terms of scalability and throughput characteristics. Moreover, we summarize a list of generalizable and actionable guidelines for future blockchain-assisted projects and integration models.

## Methods

### Capabilities of Blockchain to Support Identity Management

According to the draft on Blockchain Technology Overview by the National Institute of Standards and Technology (NIST), blockchain is an immutable digital ledger system implemented in a distributed fashion (ie, without a central repository) and usually without a central authority. There are 2 major forms of blockchain-based systems: public blockchain and private blockchain [31]. In the public blockchain, the ledger is completely decentralized among the users, and anyone can participate in the system by storing the full ledger in their own custody or by transacting data using the blockchain. The private blockchain preserves some key features of the public blockchain while compromising on the fully unregulated feature. The private blockchain is mostly tested by financial institutions, firms engaged in supply chain and logistics, health care organizations, and government entities. In both the public and private blockchain cases, users and organizations want to leverage the unique capabilities provided by blockchain technology. Previous developments and implementations of blockchains and smart contracts in telehealth were built on top of Ethereum, including teleconsultation, drug administration, and medical testing [32]. The BlockHeal framework is validated by demonstrating several use cases, including user registration and verification, patient online consultation with doctors, health care and laboratory facilities, drug counterfeiting, pharmacovigilance, language translations, and emergency facilities [33]. There are issues with scalability and security features when faced with multiserver architectures [34].

#### Decentralization

Decentralization in blockchain refers to the situation where the ledger can be maintained without the need for any regulator (in the public blockchain context) and without the need for any trusted third-party arbitrator (in the private blockchain context) [35,36]. Ideally, the public blockchain is peer-to-peer and censorship-resistant. As the public blockchain can be operated without the need for any centralized authority, any person, with the help of web-based or mobile-based technologies, can join the blockchain as a user. A person or organization can participate in the public blockchain as a full node, which means they can keep a copy of the entire blockchain and take part in making the entire system fully decentralized across the globe. A private blockchain’s decentralization is mainly for running intraorganizational or interorganizational processes without the need for a third-party intermediary. Business processes can be operated in the form of smart contracts in the blockchain that require the participating departments or organizations to set the terms and conditions upfront. Private blockchain ledgers can be stored within the organization or across multiple organizations to provide a higher level of trust in the transactions.

#### Consensus Mechanism

A consensus mechanism is basically a way to mitigate the double-spending problem and to create a single truth among all the participating nodes or organizations of the blockchain [35-37]. The consensus mechanism helps the blockchain to remain decentralized across the participating entities. The main benefit of such a mechanism is that it makes sure the current state of the blockchain is the same for all participants, each of the blocks in the blockchain is properly linked with the previous block, and all participating entities can verify the block. Popular consensus mechanisms are Proof of Work, Proof of Stake, Round Robin, etc. In the private blockchain, the consensus mechanism largely depends on the participating organizations. As the private blockchain is mainly useful to store information about operations that can impact multiple organizations, the consensus can also be based on a defined contract.

#### Immutability

The data stored in the blockchain are tamper-proof and immutable [35,37]. With the help of the consensus mechanism, the blockchain creates a single truth across all the nodes, and thus, it is computationally impossible and infeasible to alter the data that have already been stored in the earlier blocks. Each block contains some transactions, and based on those transactions, a cryptographic hash is created. That cryptographic hash is used to connect the block with the most recently verified block. When a piece of data on a block is altered, the hash gets changed and the block containing the altered data gets isolated from the blockchain. The tamper-proof and immutable feature of the blockchain can be found in both public and private blockchain-based solutions. This benefit of the blockchain increases the security of the data and digital assets on the ledger.

### Privacy Requirements in Identity Management

The technology literature tries to provide some technical solutions that can be implemented within the organization that is collecting digital identity data from users. Digital identity management can be a pervasive infrastructure that helps an organization handle user account provisioning, authorization, and authentication. Windley [29] emphasized the inclusion of 5 building blocks (process architecture, data architecture, technical reference architecture, policies, and interoperability framework) in the identity management system to provide better protection against any breach. However, these technical solutions cannot single-handedly solve privacy breach incidents. Thus, there are some external policies created by governments and standardization bodies in place for organizations. There are 3 categories of regulations and policies an organization might consider following. The first category involves global-level policies such as the recent General Data Protection Regulation (GDPR) and OECD’s Data Protection and User Control for Identity. The second category involves country-level policies such as The United States Privacy Act, The Swiss Federal Law on Personal Data Protection, and the CSA Model Code for the Protection of Personal Information. The third category involves general business-specific policies such as the Health Insurance Portability and Accountability Act.

Most of these regulations help an organization to define the boundary of the operation needing users’ private data and to synchronize the technical solution with regulatory compliance requirements. Ayed [38] stated that increasing users’ control over their digital identity is a key factor in decreasing the chance of privacy-related breaches such as privacy invasion and fraud. Ayed [38] further posited that an increase in control will give users ownership over their digital identity and thus will allow users to see what attributes are stored online, how those can be used further, and what steps to take for reducing the risk of becoming an unfortunate victim. To achieve this feature, Ayed [38] proposed a digital identity–related privacy requirement (DIPR), which can be used in the process of designing digital identity management systems. The DIPR includes 10 requirements as shown in Multimedia Appendix 1.

### DSR Methodology

**Figure 1:**
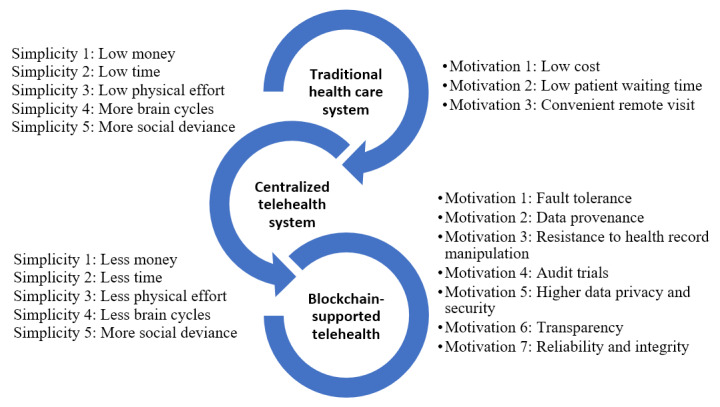
Behavior changes from traditional health care to telehealth and blockchain-supported telehealth.

DSR involves the construction of a wide range of sociotechnical artifacts [39], and the impact of this research is witnessing a more striking influence with the appropriate and effective consumption and production of knowledge throughout the research process. This paper follows the workflow toward a research contribution that can benefit software developers and technology innovators and most importantly the researchers working in this field in terms of the embedded phenomena in blockchain-assisted telehealth. The application domain of this paper is telehealth, and we argue that the solution maturity is low, while the problem space of telehealth is relatively high. Thus, based on the DSR paradigm identified in [39], we are designing a solution to improve current technical adoption. According to the Fogg Behavior Model (FBM) [40], there are 3 factors that control behavior change, namely, motivation, ability, and triggers, which can be applied to the health care domain. Specifically, to design a telehealth system and platform and promote the adoption of such a system or platform, users should be sufficiently motivated and have the ability to perform the desired behavior expected by the system or platform, and there are triggers to prompt users to perform the behavior. All 3 factors, according to the FBM, must be present at the same instant for the behavior to occur. We have adopted this behavior design model and proposed a user-centric model for the blockchain-assisted telehealth platform. Combined with the FBM theory, we present this DSR methodology to reflect the user’s motivation and ability to perform telehealth behavior and receive telehealth services triggered by both the blockchain-assisted platform and the user’s medical condition, as well as the health care service provider’s technical capability. Considering the differences between traditional, centralized, and blockchain-supported telehealth and telemedicine systems [41], we illustrate that the motivation for technology innovation adoption plays an important role in terms of user experience (UX) and acceptance aspects (Figure 1).

We have presented both the motivation aspect and simplicity/ability aspect of the behavior change from traditional health care to telehealth and blockchain-supported telehealth. With added motivation and added simplicity, our blockchain-supported telehealth platform design adopts a user-centric methodology and provides a straightforward process for the technology transformation of the underlying architecture implementation. The following section will illustrate how we designed each development cycle and present the main contributions from the design science perspective.

#### Design Cycle Description

**Figure 2:**
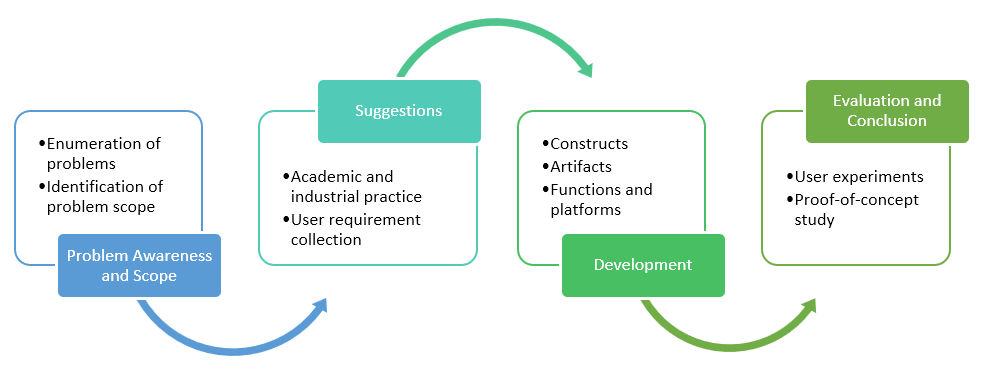
Our design science research approach.

Following the modeling design process theory [42], the overall design cycle adopted in this paper starts with problem awareness and scope, followed by suggestions from both academic and industrial practices (Figure 2). Considering the user-centric design paradigm, more than purely technical factors, this paper adds a new dimension in the second stage, which is user requirement collection. Following that, we have developed innovative IT artifacts to provide the functions and platform implementation. As such, we have presented overall DSR outcomes and contributions as follows, which distinguish this paper from previous work.

While there are applications available to support telehealth services, there are few guidelines for decentralized implementation. Based on blockchain characteristics and the needs associated with telehealth services, we have proposed a set of smart contract–driven telehealth DPs from the design requirements collected from user experiments and academic or industrial initiatives.

We have designed and presented 2 artifacts that can facilitate both user end (frontend) and backend system integration with blockchain technology, namely, the user credential management function and the access control function, which can be driven by underlying smart contracts. The proof-of-concept project demonstrates the feasibility and verifies the usable potential of the proposed concept and design theory. We have followed the reporting guidelines suggested by [43] and have provided a checklist in Multimedia Appendix 2.

From the design of the artifacts, we have presented a reflection of the design and performed an evaluation of the proposed design in terms of security properties and functionality performances, followed by a summary of the lessons learned and contributions to DSR theories.

#### Organizational Setting

In our examination of the current landscape of telehealth, we analyzed both academic literature and practical applications across the industry. Our findings indicate that the telehealth sector is in the early stages of both development and adoption. A range of functionalities have been explored in prior research, including access to medical data, processing of medical services, video conferencing capabilities, epidemiological reporting, support for diagnostics and treatment, aggregation of patient data, scheduling of visits, ordering of medications, and integration of payment and fundraising systems [44]. This study focuses on the identity management and communication aspects of telehealth services.

#### System Details and System in Use

This study selected 2 features, which patients care the most about according to our user study related to security and privacy, including the user credential management functionality and access control functionality. The user credential management functionality has a design that incorporates user motivation as well as the ability to adapt to new technologies or platforms. Meanwhile, the access control functionality follows the principle that users are respected in terms of their privacy and data security.

#### Participants

There are 2 categories of participants interviewed during the design requirement collection process, namely, general users (patient side) and subject matter expert users (health provider side). The research included data from 20 general users and 5 expert users within the United States and Europe, and the demographic characteristics of these respondents are shown in Multimedia Appendix 3. On average, the interview with general users took 71.06 minutes and that with expert users took 66 minutes.

#### Outcome and Evaluation Criteria

From the design of the artifacts, we have presented a reflection of the design and performed an evaluation of the proposed design in terms of security properties and functionality performances, followed by a summary of the lessons learned and contributions to DSR theories. Specifically, we have assessed performance metrics, such as transaction throughput, smart contract execution time, and block generation time. Our security analysis evaluated the resilience of the system against common attacks prevalent in telehealth systems.

#### Methods for Data Acquisition and Measurement

We have adopted a user-centric design approach and have started with user requirement collection. We have used the information in the report by Chanson et al [45] for developing the requirements. With the analysis of the collected data, we have moved forward to design the system functionalities that can facilitate user requirements, thus promoting user behaviors toward the telehealth platform. The purpose of the interview study is to inform the design process in terms of user requirements from both general users and expert users for a privacy-preserving telehealth solution. The interview questions are related to prior experience of use or potential functionality expectations from a telehealth artifact.

#### Methods for Data Analysis

In the development of design requirements, we have employed qualitative methods to analyze responses from our interviewees. We have used a thematic analysis approach to interpret the interview data. Our first step involved transcribing the interviews and reading the transcripts multiple times to gain a deep understanding of the content. We performed initial coding in an inductive manner, where codes were generated from the data without trying to fit them into a pre-existing coding frame. This allowed emerging themes to be grounded directly in the interview data. After coding, we grouped related codes into potential themes. These themes were continually reviewed and refined to ensure they accurately represented the data. This part of the analysis was iterative, as themes were defined and redefined through discussions among the research team members. Each theme was then reviewed in the context of the coded extracts and the entire data set. This was to ensure that themes were coherent, consistent, and distinct. We finalized themes by defining and refining their specifics and how they are related to the user requirements in telehealth. This step involved a detailed analysis of each user requirement captured.

In the system implementation section, following the design evaluation guidelines proposed in [30], we have evaluated our artifacts using both the descriptive evaluation method and analytical evaluation method for the security domain, and the experimental evaluation method for the performance domain.

### Ethical Considerations

We did not submit an application for ethics review board assessment because our research methodology has been classified as “Quality assurance activities designed to continuously improve the quality or performance of a department or program (results will not be shared with public)” according to the Office of Research Integrity (ORI) at Florida International University [46]. The interview data were collected anonymously and contained no information that could be linked to specific individuals. The data collected in this study were primarily used to design a software architecture integrated with blockchain technology, following a quality improvement approach. The raw data were analyzed to extract user requirements, which informed the design of the system architecture and the implementation of system functions. We used the raw data solely for the purpose of designing system functions. This paper presents the resulting system architecture and functions, rather than the original data, ensuring that specific details from the interviews remain confidential and are used solely for system development purposes. The results presented in this paper are derived from our analysis and not the original data.

## Results

### Developing Design Requirements for Telehealth Artifacts

#### User Requirements (Frontend)

**Figure 3:**
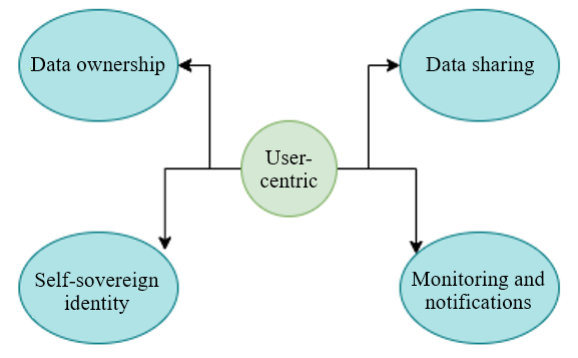
User-centric approach for requirement collection.

From the patient or user-centric standpoint, we retrieved the following 4 features from the survey as design requirements: data ownership, data sharing, self-sovereign identity (SSI) capability, and system monitoring with user notifications (Figure 3).

##### Data Ownership

Ownership means that everyone using the platform owns their data. They can limit personal information exposure at their own control. Fragmented records are easy to merge and integrate without any third-party involvement. Patients can own their health records, such as telehealth visits and immunization records, for future reference or verification. We have provided snippets of interview comments that helped us generate the data ownership requirements.

One current telehealth user mentioned:

Telehealth should make sure, each time before the user who logs into the system, the system itself should at least ask the person one or two personal questions, which have been asked before, so that it could verify if that is it the very same person who is logging in.

Another telehealth user commented:

Perhaps telehealth systems should send me regular updates regarding the security of my personal data and should allow me to have control over how secure my data is and what security measures are put in place to protect it.

One expert user mentioned:

I would want patients to be able to access their data if they wanted to, so that they can see what is recorded about them. I would want there to be a clear way for patients to review the data and communicate with the service if they felt any information was incorrect or needed amending. I would want patients to be offered clear choices about how their data is managed.

##### Data Sharing

The patient can decide what information gets shared and to whom the information gets shared. The permission for data sharing does not rely on health care providers or medical institutions, which means that the reliance on institutions is minimized. Most importantly, the patient can only share the necessary information without revealing personal or sensitive information. We have provided several interview comments on the data-sharing requirement of telehealth.

A user commented:

In my opinion, information should only be shared between doctors - obviously - but also only when completely necessary. For example, if someone is visiting a mental health professional, I think a big concern is that the information shared will be shared with their family doctor for example.

Another user mentioned:

Data that is shared between the medical services should only be found on the patient history, only with his or her permission, so that they know that they were there ones who allowed it to happen or to take place.

A medical professional stated:

There is a vast security network in place to allow the safe sharing of data between medical service providers. I would expect the telehealth system to have similar security systems in place. For instance, there should be levels of clearance given to different members of staff as to which data they are able to access and who is able to share it.

##### SSI Capability

Patients are required to verify their identity each time they request a telehealth visit. The identification and verification processes are launched on top of the blockchain-supported credential authentication platform. The verified credentials will be hashed and recorded in the blockchain ledger and the patient’s local storage for immutability and protection purposes. We have provided interview comments that helped us generate the SSI requirements.

One current telehealth user mentioned:

Telehealth systems can verify user identity by asking questions only the specific user would know the answers to. Identity verification software can be researched and utilized.

Another telehealth user commented:

I think it would be beneficial for video calling to be the main method of telehealth services, and then the patient would be able to display their health card.

An expert user agreed:

The person could also be asked to input some verification details when logging on for the first time - for example, they could be asked to put in their name, date of birth and address, and these could be checked against the recorded details - if these did not match, then the person would not be able to log on.

##### Monitoring and Notifications

The patient has the option to enable the function of monitoring risks according to their health conditions after the telehealth visit, with assisting wearable devices or other medical equipment. The patient can also be notified about safety measures or potential risks of certain activities, especially in the pandemic era, with their permission and initiative to share the intended activities. We have provided several interview comments on the monitoring and notification requirements of telehealth.

A user commented:

Telehealth systems can monitor my progress by allowing me to enter symptoms, information, personal notes, and observations, and so forth into an online diary or sorts.

Another telehealth user mentioned:

I think the best option would be to follow up via the same method as the initial consultation that allows for either user to communicate whenever they are available rather than having to be connected at the same time.

An expert user stated:

I would like optional questionnaires on the system, that the patient could be asked to complete during or after each session. I would like there to be a broad range of options available, so that the patient and I could pick the questionnaires most suited to them and their health conditions, and we could pick the frequency of monitoring this.

#### System Functions (Backend)

**Figure 4:**
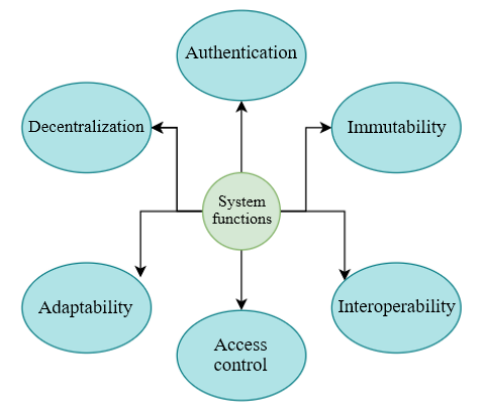
System functionality analysis.

For system functions in the telehealth scenario, we have considered 4 aspects, including architecture, data operation, integration, and interoperation among systems, which will be improved for the adoption of the blockchain component. Moreover, for the security of user accounts and access management, the system functions will include authentication and access control. Detailed descriptions of each function are presented below (Figure 4).

##### Decentralization

The system will be built on top of decentralized architecture, which removes the vulnerability of a single point of failure and removes the reliability on a trusted third party. With decentralized architecture, there is extra assurance of the resilience of the system, especially when faced with potential attacks from both insiders and outsiders. The decentralized governance of the data and system operations assures that the system still functions normally even when some portion of the system is compromised. For telehealth visits, it is essential to maintain the business uninterrupted during the communication process between patients and health care providers, and maintain after-visit accessibility. The decentralized server architecture will provide seamless communication and connection whenever the patient needs access to either service or data remotely.

##### Immutability

The blockchain structure provides the function of immutability and tamper resistance in terms of data protection and makes sure that any data records or access logs cannot be modified by malicious users. Both the data records and access records are hashed and stored in the blockchain and local storage. This technique can prevent both insider and outsider attacks. Further, it is essential to provide the authenticity of system logs when auditing or digital forensics is requested.

##### Authentication and Access Control

As we mentioned in the user requirements, the SSI mechanism has been implemented with blockchain technology, so the authentication process in the system is based on the identity and user credentials provided when requested. Delegated access can be designed for users at different levels so that only authorized users can access the data, and the data can only be accessed by users authorized with delegated access and valid tokens [47]. Once the tokens expire, access is revoked. Appropriate access control mechanisms should be designed and implemented to protect data security and defend against attacks such as email phishing and social engineering attacks.

##### Adaptability

This system function is required at 3 different levels. First, the telehealth system should adapt to user groups (pediatrics, adults, and seniors). The sign-up process should be easy and transparent. There should not be any technical barriers for users to adopt the system. Moreover, system access cannot be slow and costly. Users should be capable of accessing their comprehensive medical records at their fingertips [48]. Second, the telehealth system was demanded by the pandemic situation, but it should also adapt to postpandemic use cases when the telehealth need will be further developed and improved [49]. Third, for compliance with regulations, policies, and guidelines from federal, state, and local governments, the system should be accompanied by a function to adapt well to future updates and upgrades by continuous system iterations.

##### Interoperability

The artifact considers and integrates the system upgrade with the function and capability of interoperability, considering the current health care system separation and data silos. The blockchain component serves as an underlayer of the telehealth system and should be designed as an open application programming interface for easy adoption and interoperability. Users should be capable of accessing their medical records from other systems, which can be easily imported. The transfer of records should be seamless, saving money and time.

### Deriving Blockchain DPs and Design Features for Privacy Protection

Following the existing body of knowledge and the design requirements concluded from the previous step, we have derived the following basic DPs that can be fulfilled with design features [50] summarized in the next step.

First, considering the objectives of this research, the UX and DSR paradigms can be combined to design a user-centric IT system [51] and can offer telehealth services to potential users. By applying the DSR and UX principles and exploring how these 2 paradigms can individually and jointly strengthen the design and development processes for user-centric systems, we can identify their value to both theory and practice. Thus, we have derived the following DP (DP1): Focus on a user-centered business process before, during, and after the telehealth service is provisioned. The second philosophy during the design was a minimalist design, and it is summarized as the “less is more” principle, due to the reality that irrelevant information distracts [52]. This DP reflects the system design requirements of adaptability and interoperability and is summarized as follows (DP2): Redesign the business process with minimal change to the current working system in telehealth services.

Owing to the business logic and nature of health care service providers, the telehealth platform needs coordination among multiple health care providers and relevant stakeholders in the telehealth ecosystem to serve many patients on demand [53]. Thus, a decentralized design pattern allows a real-time patient response in terms of workload balance. A health care member in the blockchain network can join and access the telehealth platform on demand while the number of patient requests varies. This will lead to the maximization of business value and patient benefit. Thus, we summarize the DP as follows (DP3): Increase business value and reduce costs associated with telehealth integration and adaptation. Another DP is as follows (DP4): Apply automated business process design to telehealth system configuration and maintenance. Considering the user requirements of data ownership, automation can be used to implement and accomplish data sharing, identity management, and monitoring. Automation can be realized by a blockchain smart contract where a business process can be decomposed into single steps that are performed repeatedly and follow specific rules [50]. Specifically, in smart contract execution, the business process can be translated into algorithms, which a computer can execute automatically and repeatedly, in near real-time patterns. Meanwhile, automation can reduce the cognitive efforts required from human attention, thus improving the system efficiency and removing human errors. Another DP is as follows **(**DP5): Security-oriented design baked into the entire data lifecycle. Security is always a crucial component, especially when faced with sensitive patient data. The user requirements for frontend design include patient authentication and identification verification processes, which can ensure that the patient is the same person they claim to be. Meanwhile, patient data can be considered a critical asset, and the platform should strictly enforce designed access control policies so that only authorized entities can access patient data and should adopt auditing components.

Existing blockchain-supported telehealth platforms have various features that can benefit both users and health care providers, as well as other stakeholders. A summary of the features is presented in Table 1.

**Figure 5:**
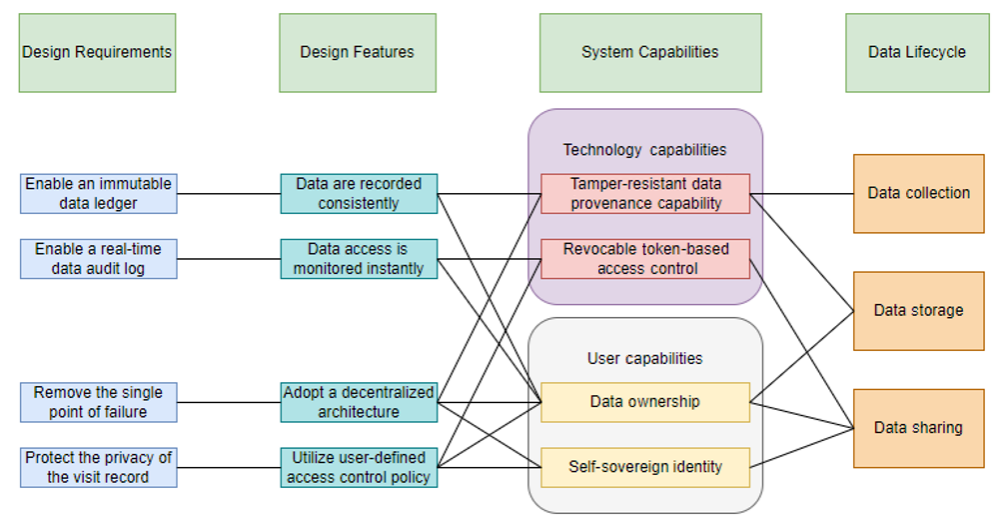
Proposed design features.

Based on the DPs, we have proposed the following design features in this paper to provide a blockchain-assisted telehealth solution (Figure 5). For the system functions in the telehealth scenario, we will consider 4 aspects, including architecture, data operation, integration, and interoperation among systems, which will be improved for the adoption of the blockchain component. Besides, for the security of user accounts and access management, the system functions will include authentication and access control. The system will be built on top of the decentralized architecture, which removes the vulnerability of a single point of failure and removes the reliability on a trusted third party. With decentralized architecture, there is extra assurance of the resilience of the system, especially when faced with potential attacks from both insiders and outsiders. The decentralized governance of the data and system operations assures that the system will function normally even when some part of the system is compromised.

For telehealth visits, it is essential to maintain the business uninterrupted during the communication process between patients and health care providers, and maintain after-visit accessibility. The decentralized server architecture will provide seamless communication and connection whenever the patient needs access to either service or data remotely. The SSI mechanism will be implemented with blockchain technology, and thus, the authentication process in the system will be based on the identity and user credentials provided when requested. Delegated access can be designed for users at different levels so that only authorized users can access the data, and the data can only be accessed by users authorized with delegated access and valid tokens. Once the tokens expire, access is revoked [47]. Appropriate access control mechanisms should be designed and implemented to protect data security and defend against attacks such as email phishing and social engineering attacks. Overall, the security design is baked into the entire data lifecycle, from data collection and data storage to data sharing.

**Table 1:** Existing blockchain solutions and design features.

Design features	Blockchain capability and components required	Benefits and improvements
Medical data access; medical records for distant consultations; patient data and prescription	Decentralized network Smart contract Tamper resistance	Patient outreach: extend the reach and expand health care services to patients Data provenance: clinical data; patient data
Medical services processing	Transparency Peer-to-peer communication Smart contract	Convenience: conveniently monitor and manage long-term patient care Workforce limitations: minimize patient access and workforce limitation issues
Video conferencing	Peer-to-peer communication Data provenance Smart contract	Effectiveness: present more effective use of health care provider’s time slots Throughput: assist physicians to serve more patients Diagnosis time: prevent delay of diagnosis for certain deadly conditions or diseases Social distance: prevent the spread of airborne infections Revenue: provide new streams of revenue to physicians and hospitals
Epidemiology reporting and remote monitoring	Peer-to-peer communication Smart contract Data provenance	Convenience: conveniently monitor and manage long-term patient care Throughput: assist physicians to serve more patients
Diagnostics support; emergency service and translation support	Peer-to-peer communication Data provenance Smart contract	Convenience: conveniently monitor and manage long-term patient care Data provenance
Treatment support and doctor recommendation	Immutability Smart contract Data provenance	Data provenance Throughput: assist physicians to support more patients
Patient data aggregation	Immutability Peer-to-peer communication Data provenance Smart contract	Identity management Access control: authorize participants without a central authority
Visit arrangement	Peer-to-peer communication Smart contract	Convenience: conveniently arrange patient care Throughput: assist physicians to serve more patients
Medicine ordering and drug tracking	Immutability Data provenance Smart contract	Convenience: conveniently monitor and manage long-term patient care Data provenance
Automated payments for billing and claims	Immutability Data provenance Smart contract	Automated execution: payment settlement Data provenance Immutability: ledger service
Fundraising	Data provenance Smart contract	Identity management: manage user identities Anonymity: preserve patient anonymity Immutability: ledger service

### Developing and Implementing the Blockchain Platform for Privacy Protection

This paper discusses 2 features that are implemented and that users directly interact with according to our user study, namely, user account/credential management functionality and token-based access control functionality, both of which are closely related to security and privacy.

**Figure 6:**
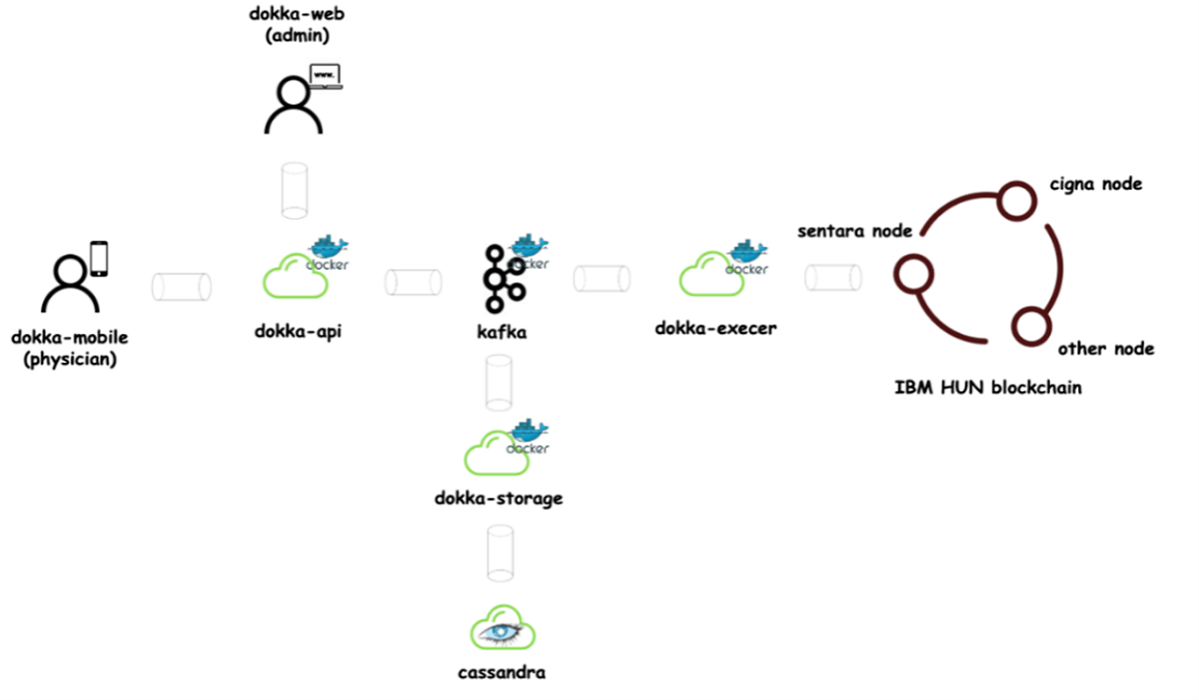
Dokka platform’s microservice-based architecture.

To provide smart contract–driven functionalities, we have built our platform, Dokka (Figure 6), with the Rahasak blockchain [54]. Rahasak is a permissioned blockchain platform where participating institutions can register and enroll their identity through a membership management service. The platform has been designed using a microservices architecture. Dokka is a blockchain SSI-based credentialing engine for telehealth platforms. Multiple health care companies (eg, Sentara and Cigna) collaborate with blockchain platforms (eg, IBM HUN). There will be a separate blockchain node for each health care company. Smart contracts will be running on each blockchain node. The data on the platform can be shared among multiple hospitals in the network.

#### Smart Contract–Supported User Credential Management

**Figure 7:**
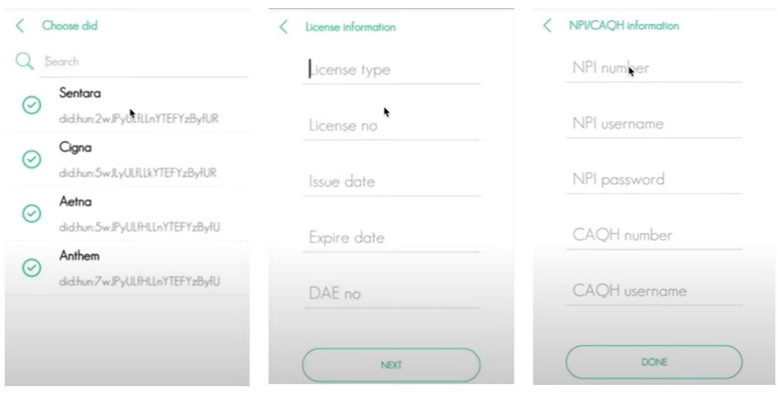
Physician interface for credential management.

**Figure 8:**
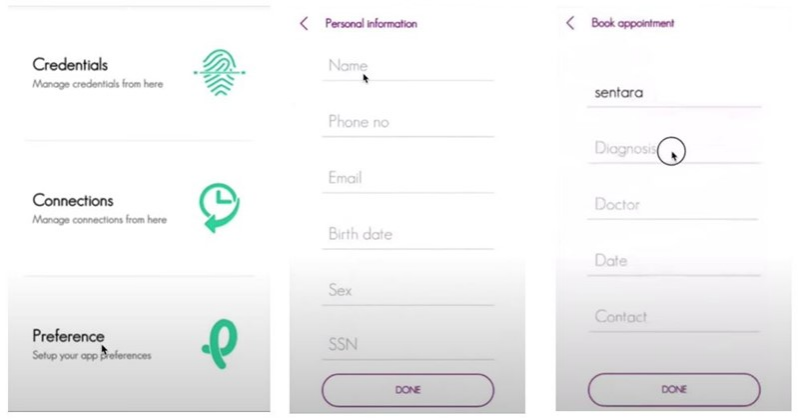
Patient interface for credential management.

Physicians can use this application to manage their physician credentials (Figure 7). Patients can use the application to manage their telehealth functions such as appointment booking and prescription handling (Figure 8). An SSI-based identity wallet has been implemented to capture or verify user identity proofs. It stores user data on the blockchain platform by using SSI proofs. All services are dockerized and available to deploy with Kubernetes [55]. The Dokka platform is built on the microservice architecture [56] to support high scalability and high transaction load following the single responsibility principle. To cope with high transaction loads and back-pressure [57] operations in practical systems, we have followed a reactive stream–based approach in the implementation process. All microservice communications are handled via the Apache Kafka message broker. The functionalities of the blockchain have been implemented with Aplos smart contracts. There are 3 main smart contracts: account contracts, device contracts, and credentialing contracts. The Dokka mobile wallet is the client application on the Dokka platform. The functions implemented in the blockchain smart contracts will be invoked by mobile clients. The requests generated from mobile apps will be directed to blockchain smart contracts via the Dokka gateway service. There is a peer-to-peer communication channel between Dokka mobile wallets to exchange credential data.

#### Token-Based Access Control

A missing design component in existing studies is the access control mechanism in telehealth platforms. This project adopts token-based access control to secure personal health data access and exposure. The access control policies are enforced by blockchain smart contracts in a decentralized way to ensure integrity and remove the necessity of a trusted third party. Various stakeholders can request data access from the data owner, that is, the registered user in the system. Users have the option to grant, deny, and revoke access from other participants. User registration is based on U-Prove technology [58], which includes 3 entities, namely, issuer, prover, and verifier. In our implementation, the issuer and verifier represent the same entity. Parameter definitions for both the prover and issuer in the token generation phase are listed in Table 2.

**Figure 9:**
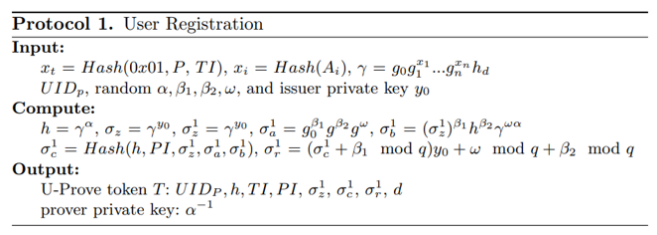
Access token generation algorithm.

With the above information provided, we have used issuance protocol version number 0x01 and the user platform identification key to generate a private key in the token generation protocol (Figure 9). For each telehealth request, the user will generate an access token for the requester. The algorithm supports the generation of multiple keys for a single user for better performance. The Dokka platform nodes issue tokens to users with a digital signature. For privacy concerns, the application attributes are hashed for the generation of U-Prove–based tokens. During certain circumstances, the issuer can generate multiple tokens at one time for better performance.

**Table 2:** Parameter definitions for both the prover and issuer in the token generation phase.

Parameter	Parameter definition
*(TI): TI* *∈* (0, 1)*	The value of the token information field
*(PI): PI* *∈* (0, 1) *	The value of the prover information field
*(AA): (A1, ..., An), TI*	Application attributes
*UID*_*H*_	Identifier for the secure hash algorithm
*(g0, g1, ..., gn, gt)*	Issuer’s public key
*(e1, ..., en)*	Format of each application attribute
*(IP): UIDp, desc(Gp),UID*_*H*_ *,(g0, g1, ..., gn, gt),(e1, ..., en)*	Issuer parameters
*P = H(IP)*	The hash of the *(IP)*
*(DP): gd, xd, hd*	Device parameters

### Evaluation of Security and Design Performance

#### Security Analysis

From the analytical perspective, our security design is baked into the overall telehealth architecture. Based on the attack surface analysis of the telehealth platform, we have considered the authentication, communication, and auditing processes. According to the design blueprint for a large-scale telehealth platform [53], 5 layers are taken into account. Using smart contract–driven methodology, this paper improves the security level for each layer, including user authentication, user credential verification, access control, data protection, and auditing (layers 1-5). Users interact with layer 1 directly, and this is the initial step for the telehealth service. During this phase, users are authenticated by the blockchain network via an SSI-enabled identity verification process. Layer 2 determines the appropriate access control policy, and the tokens are issued in this step for both patients and stakeholders. Specifically, the tokens are periodically refreshed to defend against replay attacks, man-in-the-middle attacks, stolen verifier attacks, and impersonation attacks [34]. Layer 3 functions on top of the issuance of the tokens, and access is granted automatically via smart contracts. Users can perform data-sharing operations with other parties in layer 4 with appropriate cryptographic settings. The auditing process in layer 5 monitors all the activities in this process and automatically generates alerts once a malicious behavior is detected. The auditing process is captured by smart contracts so that the log is tamper-resistant.

As pointed out in [53], the user registration and identification verification layer is the frontend layer, which is a representative artifact of user-centered design. Thus, we have analyzed the security properties provided by the user registration and identification verification process here from a descriptive perspective. The critical identity information could be accessed by attackers via technical approaches such as key compromise and identity theft. Impersonation attacks are typical cases of identity manipulation attacks, which can be launched via identity theft or credential compromise. If attackers get access to previous account tokens, they could recognize or identify a pattern from these tokens, and thus, valid tokens could be compromised eventually.

For the token-based access control component, there are potential attacks such as replay attacks. Smart contracts will provide preventive measures against token-related attacks by the enforcement of automatic program execution and peer validation of tokens. In this way, the replay attack will fail due to unauthorized access and the consensus protocols designed for peer validation. For the video conferencing component, there are potential attacks, such as man-in-the-middle attacks and impersonation attacks, but the peer-to-peer communication process is well protected by the underlying smart contracts and cryptographic algorithms.

In summary, the proposed design provides resistance against common attacks targeting general user communication processes with multiple stakeholders involved. Specifically, the design provides resistance against replay attacks, collusion attacks, man-in-the-middle attacks, stolen verifier attacks, and impersonation attacks [34].

#### Performance Analysis

Technically, the blockchain can support telehealth platform functionalities; however, previous studies have mentioned that the blockchain’s scalability is currently limited [59]. We evaluated the performance in terms of transaction throughput, smart contract execution time, and block generation time. Besides, the scalability and throughput of blockchain transactions have always been concerns and bottlenecks for system processing capability. In the following experiments, we will investigate these different aspects of system performance and compare them with existing solutions. There are 3 Kafka broker nodes for consensus management, with 3 Zookeeper nodes representing service providers in Dokka with multiple Rahasak blockchain clusters in AWS 2xlarge instances (16 GB RAM and 8 CPUs). The simulation platform uses Apache Cassandra as the state database and is implemented with Scala functional programming and the actor-based Aplos smart contract platform. The evaluation results are obtained with a varying number of blockchain peers in different evaluations.

**Figure 10:**
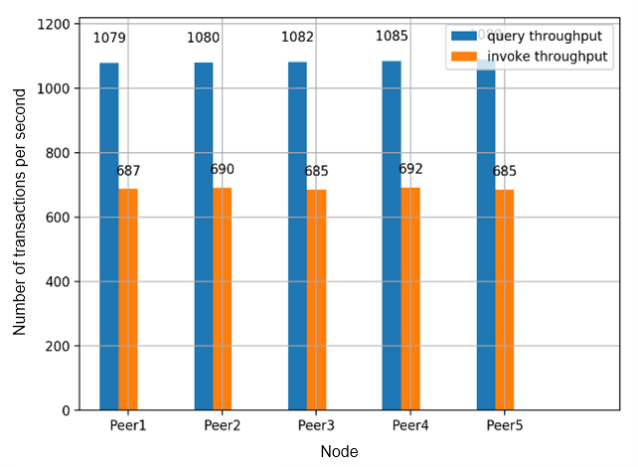
Invoke and query transaction throughput.

**Figure 11:**
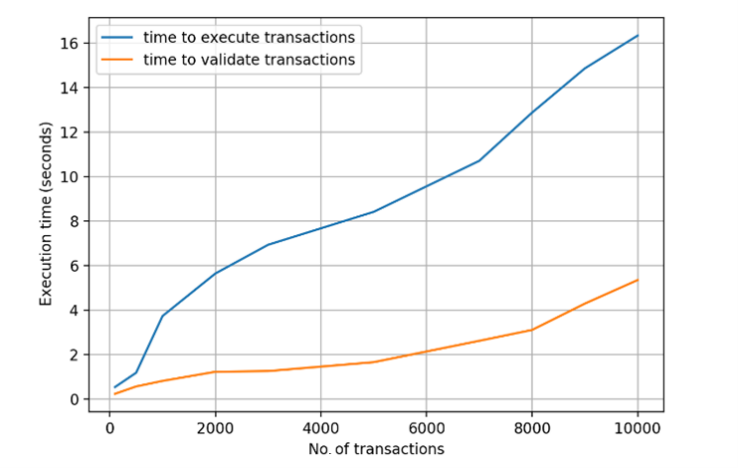
Transaction execution time and validation time.

For transaction throughput, we recorded the number of digital identity proof create transactions and digital identity proof query transactions that can be executed in each peer in the Dokka platform. Digital identity proof query transactions and invoke transactions generate transaction records that update the status of the ledger, while query transactions only perform search operations without updating the existing transactions. We obtained consistent throughput in each peer on the Dokka platform, flooded with concurrent transaction load (Figure 10). Similarly, we recorded the time cost to execute and validate different sets of transactions (Figure 11), which indicates an acceptable and linear time overhead.

**Figure 12:**
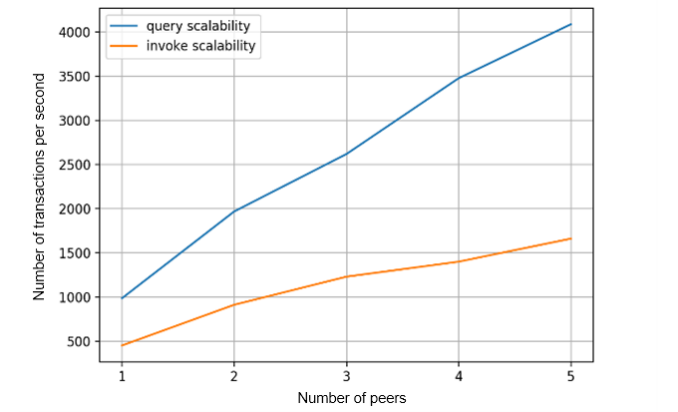
Transaction scalability.

**Figure 13:**
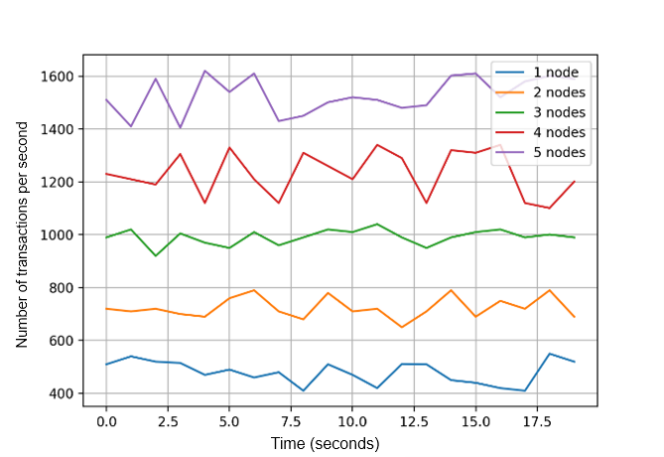
Transaction execution rate.

Figure 12 shows the transaction scalability aspect. As the number of nodes in the cluster increases, the transaction throughput increases linearly. Query transactions have higher scalability than invoke transactions since the query operation avoids ledger status updates. To evaluate the transaction execution rate, we captured the number of transactions executed using different numbers of blockchain peers. Figure 13 shows that when the number of peers increases, the transaction execution rate increases proportionally.

**Figure 14:**
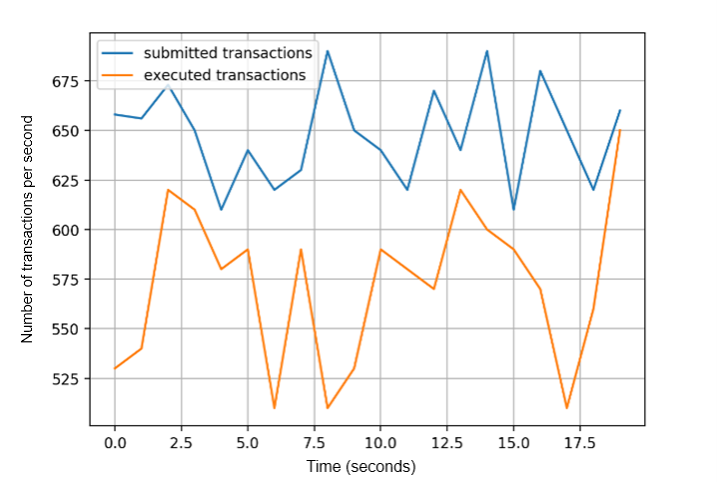
Transaction execution rate and submission rate in a single blockchain peer.

Figure 14 shows the transaction execution rate and transaction submission rate in a single peer. The results are satisfying with regard to the stable performance over time. Moreover, the average time to generate new blocks is recorded against the number of transactions in a block. Block generation time depends on the message propagation time, block hash time, and transaction verification time. When the number of transactions increases in a single block, these factors will increase. Therefore, when the number of transactions increases, block generation time also increases correspondingly. As shown in Multimedia Appendix 4, it takes 8 seconds on average to create a block with 10,000 transactions.

## Discussion

### Principal Findings

According to the matrix approach [39] proposed for evaluating the knowledge contribution in DSR from the information system discipline, the overall contribution in this paper falls into the advancement quadrant introducing an innovation. We presented a decentralized telehealth platform design and development approach that can help guide future blockchain integration and new technology adoption. We have summarized the blockchain telehealth design implications (DIs) as our knowledge contribution to the design theory for blockchain integration strategies.

#### DI1: Prioritize User Requirements in the Business Process Design

Try to focus on the human experience and consider the actions and needs of patients who will use the portal and not just focus on the technical aspects of the system design. Recognize the importance of collaboration with patients to ultimately deliver an impactful experience. User-centric design will benefit every stage of the research, from testing and validation to solutions, in order to meet user needs. Consumer preferences will play an important role in the transition from the pandemic period to the postpandemic period [60]. The blockchain integration process is hidden from users, and most of the time, users are not required or not aware of their actions to interact with blockchain networks. Thus, when designing smart contracts, try to optimize the workflow with user-centered principles and provide maximized simplicity.

#### DI2: Redesign the Business Process With Minimal Change

Business processes are not standalone; thus, it is very important to reuse the components of blockchain integration and share the common design artifacts across the system development lifecycle. For health care providers, there is always a need to minimize costs without reducing the quality of the services provided to customers [61].

#### DI3: Increase Security and Business Value

Security design should be baked into the entire lifecycle of telehealth design. Specifically, data management and access control policies should be enforced across the system components involved in patient data operations. Along with deployability, scalability, reliability, and ease of use, patient trust should be taken care of so that users are motivated to adopt the telehealth platform at their comfort level. Do not oversimplify the process, and try to understand the business value up front and focus on long-term benefits.

#### DI4: Pilot Project Experiment and Iteration

End users can provide feedback about the current iteration of the portal to inform future improvements that will meet their needs. Considering complicated patient demographics and enabling various functionalities in multiple rounds of iterations will boost patient enrollment and engagement [62]. Evaluate different platform options and product features to best suit the business use cases for telehealth innovations.

### Limitations

Blockchain as a new technology is not yet fully developed in the health care domain [63] or framed by government rules and regulations [64]. There are some technical limitations of our prototype, especially related to the health care domain. First, generalized and standardized mechanisms for institutional collaboration are required to address the gaps between different medical procedures before, during, and after the telehealth process. Second, the sharing of health care data can lead to unintended intellectual information disclosure if the deployment of the system is not done correctly. Using thorough planning and negotiation between institutions can address this limitation. Third, the incentive mechanism for institutional collaboration could also be explored to address this limitation in future work. Full participation from all stakeholders [65] is extremely essential for promoting the adoption of this innovative architectural design to achieve better health care delivery.

### Comparison With Prior Work

Prior work has provided an overview of the adoption of digital technologies in the health care domain, which highlighted blockchain as an innovative solution to provide assistance to diagnosis during a crisis [66]. The necessity of security regulations and privacy policies is indicated to adopt blockchain technology [41], which calls for studies like ours to protect patient data and especially the communication process in the telehealth platform. Previous developments and implementations of smart contracts were built on top of Ethereum for 3 telehealth services, including teleconsultation, drug administration, and medical testing [32]. The BlockHeal framework has been validated by demonstrating several use cases, including user registration and verification, patient online consultation with doctors, health care and laboratory facilities, drug counterfeiting, pharmacovigilance, language translations, and emergency facilities [33]. There are issues with scalability and security features when faced with multiserver architectures [34]. We have compared blockchain-assisted telehealth solutions in Table 3 and have highlighted our contribution.

In summary, while there are applications available to support telehealth services, there are few guidelines for decentralized implementation. Based on blockchain characteristics and the needs associated with telehealth services, we have proposed a set of smart contract–driven telehealth DPs from the design requirements collected from user experiments and academic or industrial initiatives. We have designed and presented 2 artifacts that can facilitate both user end (frontend) and backend system integration with blockchain technology, namely, the user credential management function and access control function, driven by underlying smart contracts. The proof-of-concept project demonstrates the feasibility and verifies the usable potential of the proposed concept and design theory. From the artifact design, we have presented a reflection of the design and have performed an evaluation of the proposed design in terms of security properties and functionality performances, followed by a summary of the lessons learned and contributions to DSR theories.

**Table 3:** A comparison of blockchain-assisted telehealth solutions.

Solution	Storage	Blockchain role	Smart contract	Blockchain type
Authentication for multiserver edge computing [34]	Edge and cloud	Authentication and key agreement	Practical byzantine fault tolerance (PBFT)	A lightweight consortium blockchain
MEC^a^ real-time authentication between IoT, MEC, and cloud [67]	Storage offloading capability	Rewarding scheme	Access control	Permissioned, Hyperledger Fabric
Blockchain-based telehealth framework, BlockHeal [33]	Decentralized Hyperledger Fabric	Decentralized applications (DApps)	Central health regulatory authority	Permissioned, Hyperledger Fabric
Remote patient monitoring [68]	EHR^b^ data management using Ethereum	Blockchain-oriented software (BOS) engineering	Solidity	Permissioned, Ethereum and Hyperledger Fabric
MedHypChain [69]	Cloud blockchain	Identity-based broadcast group signcryption mechanism	PBFT chaincode	Permissioned, Hyperledger Fabric
Three telehealth services [32]	IPFS^c^, cloud, and blockchain	Tracing, tracking, alert, and provenance	Public	Permissioned, Ethereum
Our solution	IPFS and blockchain	Tracing, tracking, access control, identity management, alert, and provenance	Actor-based smart contract	Permissioned, Rahasak

^a^MEC: multiaccess edge computing.

^b^EHR: electronic health record.

^c^IPFS: InterPlanetary File System.

### Conclusion and Future Directions

We presented the design, development, and implementation of a blockchain-assisted and smart contract–driven telehealth platform that can help users communicate remotely and improve medical services efficiently. The system adopts a user-centric approach and follows the behavior design model to promote new technology adoption among different groups of users. We further evaluated the security and performance of the platform, which exhibited the feasibility and efficiency of the proposed platform. Moreover, we concluded with a set of actionable guidelines and DIs to facilitate the design of a practical blockchain-based telehealth platform. Owing to the limited adoption of blockchain technology in the health care sector, there is a lack of fundamental design theory for blockchain technology integration in the current stage [44]. It is important to address who gets to see what data and when [70]. Future research can dive deep into each service model and analyze the market segmentation as a new ecosystem. The application of nonfungible tokens to health care [71] is another application scenario of blockchain technology that will significantly transform the innovation of the health care industry. For example, telehealth record data can be shared directly with the patient after the televisit and with the health care provider or insurance provider. This might lead to incentive plans that lower insurance premiums paid by patients out-of-pocket when they can demonstrate cost-efficient and flexible health care management.

This study extends the existing body of knowledge and develops a set of actionable guidelines to facilitate the design of a practical blockchain-based telehealth platform, reflecting the rigor of design research contributions. To the best of our knowledge, few studies have focused on this topic. The extension of our work will benefit several new directions for new technology integration, blockchain adoption, data privacy, and trust in multiple aspects of smart and connected health. Adapting to the sharing economy approach, the telehealth platform should explore the potential as a one-stop service application for patients in need and a promising solution for stakeholders in terms of a new business model.

## Appendix

Multimedia Appendix 1 Digital identity-related privacy requirements.

Multimedia Appendix 2 Reporting guideline checklist.

Multimedia Appendix 3 Demographic characteristics of interview respondents.

Multimedia Appendix 4 Block creation time against the number of transactions in the block.

## Abbreviations

DI: design implication
DIPR: digital identity–related privacy requirement
DP: design principle
DSR: design science research
FBM: Fogg Behavior Model
SSI: self-sovereign identity
UX: user experience

## Data Availability

Data will be provided upon reasonable request.
