# Supplementary material for: Designing A Blockchain-Empowered Telehealth Artifact for Decentralized Identity Management and Trustworthy Communication: Interdisciplinary Approach
Source: J Med Internet Res. 2024 Sep 25;26:e46556. doi: 10.2196/46556 (PMC11464941; doi:10.2196/46556)
Supplement: Multimedia Appendix 1 [file jmir_v26i1e46556_app1.docx]

**Multimedia Appendix 1.** Digital identity-related privacy requirements.

| **Category** | Requirement | Fundamental Concept(s) |
| --- | --- | --- |
| **Transparency of Identity Management** | Purpose Specification of Attributes Collection | - The purpose of the system needs to be defined. - “How the collected data will be linked to the purpose”- this also need to be stated |
|  | Openness | - The user should have control to their data. - The user should be able to see to whom their data has been disclosed. - The user will be able to challenge any mistake in data aggregation |
|  | Secondary Use | - Secondary use and sales of identity information will not be permitted |
| **Control over Digital Identity** | Consent for Attributes Usage/Release | - The user will provide consent to use or release of the identity information for the specific system purpose. - The user needs to be notified if there is any change in the system purpose |
|  | Limited Usage of Attributes | - The identity data usage only will be limited to the purpose |
|  | Limited Retention of Attributes | - The identity data will be stored for specified period |
|  | Authentication and Enrollment Needs | - The user will be able to use different authentication information for different services |
|  | Choice and Terms of the Contract | - The user will be provided with flexible terms and contracts, and they can choose not to accept parts of the contract |
| **Verifiability of Digital Identity** | Accuracy of Stored Attributes | - The stored identity data needs to accurate and updated |
|  | Compliance | - The user should be provided transparent information regarding the compliance of the requirements |
