# Supplementary material for: Designing A Blockchain-Empowered Telehealth Artifact for Decentralized Identity Management and Trustworthy Communication: Interdisciplinary Approach
Source: J Med Internet Res. 2024 Sep 25;26:e46556. doi: 10.2196/46556 (PMC11464941; doi:10.2196/46556)
Supplement: Multimedia Appendix 2 [file jmir_v26i1e46556_app2.docx]

**Multimedia Appendix 2.** Reporting guideline checklist.

| **STARE-HI item [1]** | **Item comprised in manuscript? (√/-)** |
| --- | --- |
| **Title** | **√** |
| **Abstract** | **√** |
| **Keywords** | **√** |
| **Introduction** | **√** |
| Scientific background | **√** |
| Rationale for the study | **√** |
| Objectives of the study | **√** |
| **Study context** | **√** |
| Organizational setting | **√** |
| System details and system in use | **√** |
| **Methods** | **√** |
| Study design | **√** |
| Theoretical background | **√** |
| Participants | **√** |
| Study flow | **√** |
| Outcome measures or evaluation criteria | **√** |
| Methods for data acquisition and measurement | **√** |
| Methods for data analysis | **√** |
| **Results** | **√** |
| Demographic and other study coverage data | **√** |
| Unexpected events during the study | - |
| Study findings and outcome data | **√** |
| Unexpected observations | - |
| **Discussion** | **√** |
| Answers to study questions | **√** |
| Strengths and weaknesses of the study | **√** |
| Results in relation to other studies | **√** |
| Meaning and generalizability of the study | **√** |
| Unanswered and new questions | - |
| **Conclusion** | **√** |
| **Authors’ contribution** | **√** |
| **Competing interests** | **√** |
| **Acknowledgement** | **√** |
| **References** | **√** |
| **Appendices** | **√** |

References

[1] Talmon J, Ammenwerth A, Brender J, de Keizer N, Nykänen P, Rigby M. STARE-HI – Statement on Reporting of Evaluation Studies in Health Informatics. Int J Med Inform 2009; 1: 1–9
