# Supplementary material for: Designing A Blockchain-Empowered Telehealth Artifact for Decentralized Identity Management and Trustworthy Communication: Interdisciplinary Approach
Source: J Med Internet Res. 2024 Sep 25;26:e46556. doi: 10.2196/46556 (PMC11464941; doi:10.2196/46556)
Supplement: Multimedia Appendix 3 [file jmir_v26i1e46556_app3.docx]

**Multimedia Appendix 3.** Demographic characteristics of interview respondents.

|  | **General User**  **(Patient Side)** | | **Expert User**  **(Health Provider Side)** | |
| --- | --- | --- | --- | --- |
| **Characteristic** | **Number of Respondents** | **% of Group Total** | **Number of Respondents** | **% of Group Total** |
| **Gender** |  |  |  |  |
| Female | 9 | 45% | 4 | 80% |
| Male | 10 | 50% | 1 | 20% |
| Others | 1 | 5% | - | - |
|  |  |  |  |  |
| **Age Group** |  |  |  |  |
| 18 to 35 years | 16 | 80% | 1 | 20% |
| Over 35 to 50 years | 3 | 15% | 4 | 80% |
| Over 50 years | 1 | 5% | - | - |
|  |  |  |  |  |
| **Educational Qualification** |  |  |  |  |
| Less than an Associate degree | 6 | 30% | - | - |
| Associate degree | 4 | 20% | 1 | 20% |
| Bachelor’s degree | 8 | 40% | 2 | 40% |
| Master’s degree and beyond | 2 | 10% | 2 | 40% |
|  |  |  |  |  |
| **Expert Profession** |  |  |  |  |
| Doctor, Psychologist, and Pharmacist | - | - | 3 | 60% |
| Nurse and Other Support | - | - | 2 | 40% |
|  |  |  |  |  |
