# Supplementary figures and images for: Designing A Blockchain-Empowered Telehealth Artifact for Decentralized Identity Management and Trustworthy Communication: Interdisciplinary Approach
Source: J Med Internet Res. 2024 Sep 25;26:e46556. doi: 10.2196/46556 (PMC11464941; doi:10.2196/46556)

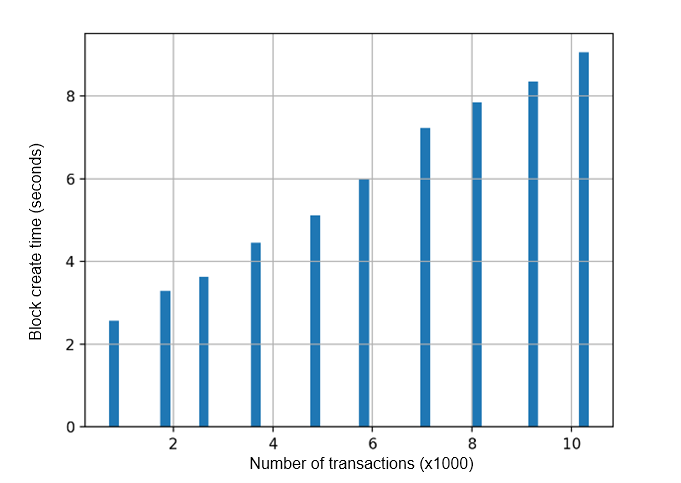

Supplement: Multimedia Appendix 4 [file jmir_v26i1e46556_app4.png]
